# The association of a frailty index derived from laboratory tests and vital signs with clinical outcomes in critical care patients with septic shock: a retrospective study based on the MIMIC-IV database

**DOI:** 10.1186/s12879-024-09430-w

**Published:** 2024-06-10

**Authors:** Huafeng Ding, Xiangquan Li, Xianjiang Zhang, Jiaqiong Li, Qinfeng Li

**Affiliations:** 1 Intensive Care Unit, Xuzhou Central Hospital, 199 South Jiefang Road, Xuzhou, 221009 Jiangsu Province People’s Republic of China; 2 Medical Laboratory, Xuzhou Central Hospital, 199 South Jiefang Road, Xuzhou, 221009 Jiangsu Province People’s Republic of China

**Keywords:** Frailty, Frailty index, Sepsis, Mortality, Intensive care unit

## Abstract

**Purpose:**

Frailty is a vulnerable state to stressors due to the loss of physiological reserve as a result of multisystem dysfunction. The physiological and laboratory-based frailty index (FI-Lab), depending on laboratory values and vital signs, is a powerful tool to capture frailty status. The aim of this study was to assess the relationship between FI-Lab and in-hospital mortality in patients with septic shock.

**Methods:**

Baseline data for patients with sepsis in the intensive care unit were retrieved from the Critical Care Medicine Database (MIMIC-IV, v2.2). The primary outcome was mortality during hospitalization. The propensity score matching (PSM) method was used to analyze the basic conditions during hospitalization between groups.The FI-Lab was analysed for its relationship with in-hospital mortality using logistic regression according to continuous and categorical variables, respectively, and described using the restricted cubic spline (RCS). Survival was compared between groups using Kaplan-Meier (KM) curves. Subgroup analyses were used to improve the stability of the results.

**Results:**

A total of 9219 patients were included. A cohort score of 1803 matched patients was generated after PSM. The analyses showed that non-surviving patients with septic shock in the ICU had a high FI-Lab index (*P*<0.001). FI-Lab, whether used as a continuous or categorical variable, increased with increasing FI-Lab and increased in-hospital mortality (*P*<0.001).Subgroup analyses showed similar results. RCS depicts this non-linear relationship. KM analysis shows the cumulative survival time during hospitalisation was significantly lower as FI-Lab increased (log-rank test, *P*<0.001).

**Conclusion:**

Elevated FI-Lab is associated with increased in-hospital mortality in patients with septic shock.

**Supplementary Information:**

The online version contains supplementary material available at 10.1186/s12879-024-09430-w.

## Introduction

Sepsis, defined as organ dysfunction resulting from dysregulation of the host response to infection, is a serious global public health problem [1, 2]. Although early detection and comprehensive treatment can improve the prognosis of sepsis patients [3, 4], the mortality rate remains as high as 40% and remains one of the most common causes of morbidity and mortality in critically ill patients [5]. Identifying new risk factors for effective intervention is essential for the clinical management of sepsis. Frailty is a syndrome characterized by a decline in the biological reserve of multiple physiologic systems [6]. Patients with frailty are vulnerable to internal or external stimuli and are at increased risk of a variety of adverse outcomes including reduced function, falls, disability, and death [7]. The frailty of critically ill ICU patients has also received a great deal of attention in recent years [8]. Research has shown that frailty status is one of the major causes of death from infection [9]. Despite the development of several frailty assessment tools for clinical applications [10], disadvantages such as the need for the evaluator to have a good knowledge base, susceptibility to subjectivity, and hysteresis limit the applicability of these methods. The physiological and laboratory-based frailty index (FI-lab) was proposed by Howlett et al. and consists of several objective laboratory tests and vital signs that can be constructed with the ease of routine clinical practice [11]. The FI-Lab has been shown to have good diagnostic accuracy and to predict clinical outcomes in a diverse group of individuals, including community residents [12], inpatient rehabilitation [13], end-stage renal disease [14], chronic obstructive pulmonary disease (COPD) [15], acute myocardial infarction (AMI) [16] and cancer [17]. No studies have been conducted on the prognostic value of FI-Lab in patients with ICU septic shock. Therefore, we sought to investigate the relationship between FI-Lab and mortality in patients with septic shock during hospitalization.

## Materials and methods

### Sources of data

The data analyzed for this retrospective study were retrieved from the database of the MIMIC-IV (Medical Information Mart for Intensive Care IV, v2.2) (Record ID: 54711406). The database contains 431,231 admission records and 73,181 intensive care unit (ICU) admission records for patients admitted to hospitals including Beth Israel Deaconess Medical Center (BIDMC, Boston, Massachusetts, USA) from 2008 to 2019. RECORD specifications were used for the present study [18].

### Participants

For analysis, We included only patients with septic shock who were admitted to the intensive care unit for the first time and to whom vasoactive drugs were applied (obtained through the visualisation view in the database) [1]. The types of vasoactive drug application included: norepinephrine, epinephrine, dopamine, and vasopressin. Patients were excluded if they met 1 or more of the following criteria: (i) age<18 years, (ii) ICU length of stay<24h, and (iii) the absence of items to construct the FI-Lab scale (n>12).

### Variates

Data were obtained from the MIMIC-IV database using the structured query language of Navicate premium 12.0.11. In addition to the items needed to calculate FI-Lab, we collected Demographic and admission information for the study population: age, sex, race, weight, length of hospital stay, survival status at the time of discharge from the hospital, SOFA, APS 3, SAPS 2, LODS, OASIS, and SIRS. information on comorbidities: hypertension, diabetes, chronic kidney disease (CKD), chronic liver disease (CLD), heart failure (HF), neurologic disease, malignancy, COPD, and acute kidney injury (AKI). Interventions received during ICU stay: mechanical ventilation, renal replacement therapy (RRT).

### The physiological and laboratory-based frailty index

A total of 33 items were used to construct the FI-Lab, including 30 laboratory tests records (24h before to 48h after the first ICU admission): blood samples (white blood cell count, platelet count, hemoglobin, total bilirubin, alanine transaminase, albumin, alkaline phosphatase, lactate dehydrogenase, urea nitrogen, creatinine, glucose, potassium, sodium, calcium, phosphorus, plasminogen time, and the international normalized ratio, activated partial thromboplastin time, fibrinogen, and troponin T), arterial blood gas samples (hydrogen potential, partial pressure of oxygen, partial pressure of carbon dioxide, and lactate), urine samples (leukocytes, erythrocytes, proteins, glucose, ketone bodies, and bilirubin), and 3 vital signs (averaged over the first day in the ICU): systolic blood pressure, diastolic blood pressure, and heart rate. Each item was dichotomized using the normal reference ranges provided in the database: a score of 0 was assigned in the reference interval, and any value outside the reference interval was assigned a score of 1. The reference value of each items are presented in Supplementary Table. For this study, FI-Lab scores were calculated by summing the words of value available and dividing the sum by the number of items included. In theory, the FI-Lab ranges from 0 to 1. The main items (n ≤$\leqslant$12) that were ultimately missing for the construction of the FI-Lab in this study included alkaline phosphatase (90%), troponin T (55%), lactate dehydrogenase (47%), albumin (43%), fibrinogen (43%), urinalysis items (30%), alanine transaminase (28%), and total bilirubin (27%).

### Outcomes

Our main concern was in-hospital mortality in patients with septic shock.

### Statistical analysis

Continuous normally distributed data are presented as means ± standard deviations (SD) and compared using Student’s t-test between groups,although skewed distributional data are reported as median and interquartile range (IQR) and compared using the Wilcoxon rank sum test or Kruskal-Wallis test. Categorical data were presented as ratios of components and analyzed using chi-square tests.

We compared FI-Lab differences (as a continuous variable)between the surviving and non-surviving groups, and to enhance the reliability of the results, propensity score matching (PSM) analyses were performed to balance the baseline characteristics between the two groups using a 1:1 nearest neighbour matching algorithm with a caliper of 0.02.Then, logistic regression was applied to study the relationship between FI-Lab and in-hospital mortality.

To ensure robustness of data analysis further sensitivity analysis was performed. FI-Lab was transformed into categorical variables (Q1,Q2,Q3 and Q4) according to quartiles. Differences between groups were compared, and the logistic regression analyses were then performed and *p*-values for trends were calculated.

In-hospital survival was assessed by applying Kaplan-Meier survival curves based on FI-Lab groupings and evaluated using the log-rank test.

Stratified and interaction analyses were applied based on age (<60 or ≥60$\geqslant$ years), gender(male or female), race (white or other), RRT (yes or no), and ventiliton (yes or no).

Finally, restricted cubic spline curves (RCS) were used to describe the correlation between FI-Lab and the risk of death during hospitalisation.

The percentage of covariates with missing data was less than 1 % for all analyses. The median was filled in for missing values of covariates. Analysis of the data was performed using Stata 17.0 software, SPSS 27.0 for Windows, and the R programming language version 4.3.1. Statistical significance was defined as a two-sided *p*-value of less than 0.05. Variables with *P*<0.05 in the univariate analysis were included in the multivariate analysis.

### Ethics statement

The studies involving human participants were reviewed and approved by Institutional Review Boards of the Massachusetts Institute of Technology and Beth Israel Deaconess Medical Center. Written informed consent for participation was not required for this study in accordance with the national legislation and the institutional requirements.

## Results

### Subject characteristics

**Fig. 1:**
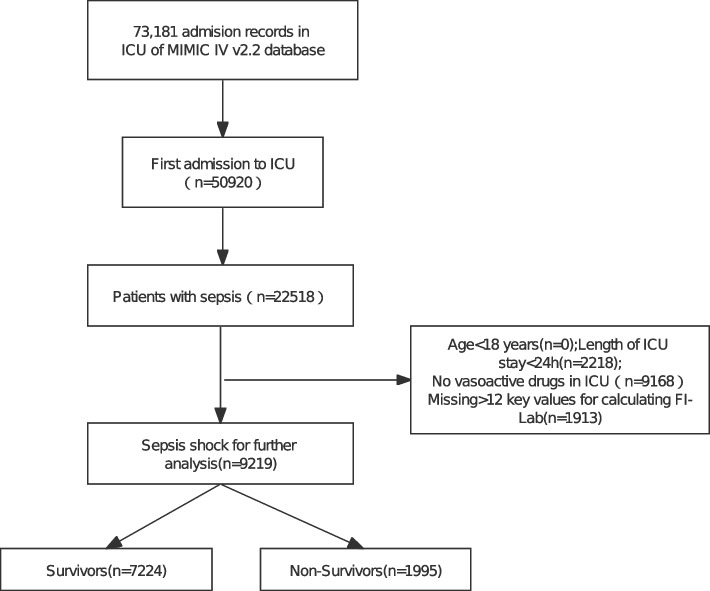
The flowchart of patient screening

We extracted data from the MIMIC-IV database for 9219 eligible patients, as outlined in the flow diagram of Fig. 1. Baseline information is shown in Table 1. 1803 pairs of patients were matched after PSM. Before PSM, FI-lab, age, gender, race, severity of illness score, RRT, and mechanical ventilation application were higher in the non-survivor group compared with the survivors, and the incidence of comorbid CKD, HF, CLD, neurologic disease, malignancy, AKI, and COPD was greater than that of the survivor group, although the incidence of hypertension in the survivor group, as well as the length of hospitalization, was higher than in the non-survivor group. There were also differences in the racial breakdown of the two groups. Whereas after PSM all variables were balanced except for length of hospital stay, and FI-Lab remained greater in the non-survivor group than in the survivorship group.

**Table 1:** Characteristics of the study population between survival and non-survival groups

Variables	FI-Lab (before PSM)		T/z/Χ^2^	*P*-value	FI-Lab (after PSM)		T/z/Χ^2^	*P*-value
	Survival	Non-survival			Survival	Non-survival		
	(*n*=7224)	(*n*=1995)			(*n*=1803)	(*n*=1803)		
Age	67.72(57.76,77.23)	70.34(58.83,80.87)	-6.844	<0.001	69.68(58.96,79.96)	70.25(58.64,80.89)	-1.004	0.315
Male (n,%)	4424 (61.24)	1101(55.19)	23.846	<0.001	1011(56.07)	1000(55.46)	0.136	0.712
Weight	82.65(70.00,98.13)	79.10(65.90, 95.50)	6.283	<0.001	79.80(67.43,96.00)	79.00(65.80,95.61)	1.152	0.249
White (n,%)	4950 (68.52)	1149 (57.59)	83.376	<0.001	1054(58.46)	1070(59.35)	0.293	0.588
Disease severity scoring system (score)								
SOFA	7(4,9)	9(6,12)	-24.293	<0.001	9(6,11)	9(6,12)	-1.597	0.110
APS3	46(34,63)	69(53,89)	-34.427	<0.001	66(50,83)	66(52,83)	-1.576	0.115
SAPS2	39(32,49)	52(42,63)	-30.56	<0.001	49(40,60)	50(41,60)	-1.854	0.064
LODS	5(4,8)	8(6,10)	-31.634	<0.001	8(6,10)	8(6,10)	-1.799	0.072
OASIS	34(28,40)	40(35,47)	-27.529	<0.001	39(33,46)	40(34,45)	-1.09	0.276
SIRS	3(2,4)	3(3,4)	-10.577	<0.001	3(3,4)	3(3,4)	-0.636	0.525
Intervention, n (%)								
RRT	690 (9.55)	559(28.02)	455.25	<0.001	438(24.29)	445(24.68)	0.074	0.786
Mechanical ventilation	5012(69.38)	1603(80.35)	92.839	<0.001	1424(78.98)	1441(79.92)	0.491	0.484
Comorbidities, n (%)								
Hypertension	3328(46.07)	722(36.19)	61.929	<0.001	663(36.77)	672(37.27)	0.096	0.756
Diabetes	2218(30.70)	590(29.57)	0.941	0.332	563(31.23)	549(30.45)	0.255	0.614
CKD	1401(19.39)	495(24.81)	28.094	<0.001	489(27.12)	453(25.12)	1.862	0.172
CLD	959(13.28)	585(29.32)	288.753	<0.001	476(26.40)	486(26.96)	0.142	0.707
HF	2168(29.90)	677(33.93)	11.280	<0.001	624(34.61)	617(34.22)	0.060	0.806
Neuropathy	873(12.08)	369(18.50)	55.127	<0.001	333(18.47)	325(18.03)	0.119	0.730
Malignancy	761(10.53)	398(19.95)	126.089	<0.001	328(18.19)	338(18.75)	0.184	0.668
COPD	539(7.46)	201(10.08)	14.468	<0.001	190(10.54)	179(9.93)	0.365	0.546
AKI	6071(84.04)	1911(95.79)	185.785	<0.001	1715(95.12)	1719(95.34)	0.098	0.755
Length of hospital stay (day)	10.72(6.48,19.02)	7.46(3.16,13.98)	19.739	<0.001	15.28(8.84,24.55)	7.72(3.39,14.14)	23.031	<0.001
FI-Lab	0.429(0.333,0.524)	0.536(0.44,0.625)	-28.362	<0.001	0.484(0.400,0.581)	0.534(0.433,0.625)	-8.661	<0.001

Abbreviations: *HR* hazard ratio, *CI* confidence Interval, *SOFA* sequential organ failure assessment, *APS* Acute Physiology Score, *SAPS* Simplified Acute Physiology Score, *LODS* Logistic Organ Dysfunction Score, *OASIS* Oxford Acute Severity of Illness Score, *SIRS* Systemic Inflammatory Response Syndrome Score, *AKI* acute kidney injury, *CKD* chronic kidney disease, *CLD* chronic liver disease, *HF* heart failure, *COPD* chronic obstructive pulmonary disease, *RRT* renal replacement therapy, *PSM* propensity score matching, *FI-Lab* The physiological and laboratory-based frailty index

### Association between FI-Lab and in-hospital mortality

In total, 1995 patients died during hospitalisation (21.64%). Whether FI-Lab was used as a continuous or categorical variable, in-hospital mortality in patients with septic shock increased significantly with increasing FI-Lab (*P*<0.001, Tables 2 and 3). The multivariate model showed that age, gender, weight, race (White), FI-Lab as a continuous variable [per 0.01-score increase: odds ratio (OR) = 1.03, 95% confidential interval (CI) 1.03-1.04, *P*<0.001], Comorbidities ( CKD, CLD, HF, neuropathy, malignancy, COPD and aki), and some disease severity scores (SOFA, APS3, SAPS2, and LODS) were independent risk factors of in-hospital mortality (Table 2). After inclusion in the multivariate model according to categorical variables (OR = 1.76, 95% CI: 1.43-2.16 for Q2, OR = 2.06, 95% CI: 1.69-2.52 for Q3, and OR = 2.91, 95% CI: 2.37-3.58 for Q4), there was a trend towards a significant increase in the cumulative odds of in-hospital mortality with increasing levels of FI-Lab (*P*<0.001), as shown in Table 4.

**Table 2:** Relationship between baseline characteristics and in-hospital mortality

Variables	In-hospital mortality			
	Univariate model (OR,95%CI)		Multivariable model (OR,95%CI)	
Age (year)	1.01(1.01,1.02)	<0.001	1.02(1.02,1.03)	<0.001
Male (n,%)	0.78(0.71,0.86)	<0.001	0.87(0.77,0.98)	0.024
Weight (kg)	0.99(0.99,1.00)	<0.001	0.99(0.98,0.99)	<0.001
White (n, %)	0.62(0.56,0.69)	<0.001	0.63(0.56,0.71)	<0.001
Disease severity scoring system (score)				
SOFA	1.19(1.18,1.21)	<0.001	1.03(1.01,1.06)	0.021
APS3	1.04(1.03,1.04)	<0.001	1.02(1.02,1.03)	<0.001
SAPS2	1.05(1.05,1.06)	<0.001	0.99(0.98,1.00)	0.007
LODS	1.30(1.28,1.33)	<0.001	1.11(1.07,1.15)	<0.001
OASIS	1.09(1.08,1.09)	<0.001	1.01(1.00,1.02)	0.087
SIRS	1.38(1.30,1.47)	<0.001	1.07(0.99,1.15)	0.085
Interventionn,n (%)				
RRT	3.68(3.25,4.18)	<0.001	1.67(1.42,1.95)	<0.001
Mechanical ventilation	1.80(1.60,2.04)	<0.001	1.37(1.18,1.59)	<0.001
Comorbidities,n (%)				
Hypertension	0.66(0.60,0.74)	<0.001	0.70(0.61,0.80)	<0.001
Diabetes	0.95(0.85,1.06)	0.332		
CKD	1.37(1.22,1.54)	<0.001	1.24(1.18,1.32)	<0.001
CLD	2.71(2.41,3.05)	<0.001	1.79(1.55,2.08)	<0.001
HF	1.20(1.08,1.33)	0.001	0.98(0.86,1.11)	0.751
Neuropathy	1.65(1.44,1.89)	<0.001	2.07(1.76,2.43)	<0.001
Malignancy	2.11(1.85,2.42)	<0.001	1.38(1.22,1.55)	<0.001
COPD	1.39(1.17,1.65)	<0.001	1.30(1.07,1.58)	<0.001
AKI	4.32(3.44,5.42)	<0.001	2.34(1.83,3.00)	<0.001
FI-Lab (per 0.01-score)	1.06(1.05,1.06)	<0.001	1.03(1.03,1.04)	<0.001

Abbreviations: *HR* hazard ratio, *CI* confidence Interval, *SOFA* sequential organ failure assessment, *APS* Acute Physiology Score, *SAPS* Simplified Acute Physiology Score, *LODS* Logistic Organ Dysfunction Score, *OASIS* Oxford Acute Severity of Illness Score, *SIRS* Systemic Inflammatory Response Syndrome Score, *AKI* acute kidney injury, *CKD* chronic kidney disease, *CLD* chronic liver disease, *HF* heart failure, *COPD* chronic obstructive pulmonary disease, *RRT* renal replacement therapy, *PSM* propensity score matching, *FI-Lab* The physiological and laboratory-based frailty index

**Table 3:** Baseline characteristics of patients after FI-Lab grouping by quartiles

Variables	Q1	Q2	Q3	Q4	P value
	(FI-Lab<0.36)	0.36≤FI-Lab<0.45	0.45≤FI-Lab<0.55	FI-Lab≥0.55	
N	2269	2244	2414	2292	
Age (year)	67.98(58.66,76.79)	68.72(59.05,77.94	68.78(58.18,79.32)	67.18(56.13,77.88)	0.002
Male (n,%)	1385(61.04)	1332(59.36)	1450(60.07)	1358(59.24)	0.587
Weight (kg)	82.9(70.0,97.3)	80.8(68.1,96.6)	81.7(69.4,97.6)	81.9(69.0,99.3)	0.048
White (n,%)	1591(70.12)	1518(67.65)	1598(66.19)	1392(60.73)	<0.001
Disease severity scoring system (score)					
SOFA	5(3,7)	6(4,8)	8(5,10)	10(8,13)	<0.001
APS3	36(29,46)	45(34,59)	55(42,71)	69(54,87)	<0.001
SAPS2	35(28,42)	39(32,48)	44(36,54)	51(41,61)	<0.001
LODS	4(3,6)	5(4,7)	7(5,9)	8(6,10)	<0.001
OASIS	31(25,36)	34(28,40)	36(31,43)	40(34,46)	<0.001
SIRS	3(2,3)	3(2,4)	3(2,4)	3(3,4)	<0.001
Intervention, n (%)					
RRT	64(2.82)	149(6.64)	349(14.46)	687(29.97)	<0.001
Mechanical ventilation	1535(67.65)	1570(69.96)	1779(73.30)	1731(75.52)	<0.001
Comorbidities, n (%)					
Hypertension	1237(56.10)	1041(46.39)	996(41.26)	776(33.86)	<0.001
Diabetes	594(26.18)	639(28.48)	779(35.19)	796(34.73)	<0.001
CKD	230(10.14)	382(17.02)	620(25.68)	664(28.97)	<0.001
CLD	141(6.21)	235(10.47)	436(18.06)	732(31.94)	<0.001
HF	562(24.77)	688(30.66)	830(34.38)	765(33.38)	<0.001
Neuropathy	355(15.65)	300(13.37)	303(12.55)	284(12.39)	0.004
Malignancy	195(8.60)	270(12.03)	336(13.92)	358(15.62)	<0.001
COPD	130(5.73)	173(7.71)	210(8.70)	227(9.90)	<0.001
AKI	1845(81.31)	1878(83.69)	2139(88.61)	2120(92.50)	<0.001
Length of hospital (day)	8.06(5.75,13.69)	9.97(5.91,17.61)	10.95(6.44,19.49)	11.84(6.00,21.07)	<0.001
FI-Lab	0.29(0.23,0.33)	0.41(0.38,0.43)	0.50(0.48,0.52)	0.62(0.58,0.68)	<0.001
In-hospital death (n,%)	171(7.54)	357(15.91)	566(23.45)	901(39.31)	<0.001

Abbreviations: *HR* hazard ratio, *CI* confidence Interval, *SOFA* sequential organ failure assessment, *APS* Acute Physiology Score, *SAPS* Simplified Acute Physiology Score, *LODS* Logistic Organ Dysfunction Score, *OASIS* Oxford Acute Severity of Illness Score, *SIRS* Systemic Inflammatory Response Syndrome Score, *AKI* acute kidney injury, *CKD* chronic kidney disease, *CLD* chronic liver disease, *HF* heart failure, *COPD* chronic obstructive pulmonary disease, *RRT* renal replacement therapy, *PSM* propensity score matching, *FI-Lab* The physiological and laboratory-based frailty index

**Table 4:** Association of FI-Lab (as a categorical variable) with in-hospital mortality

	Crude model	Model 1	Model 2	Model 3
	OR (95%CI)	OR (95%CI)	OR (95%CI)	OR (95%CI)
**In-hospital mortality**				
Continuous variable (per 0.01-score)	1.06(1.05,1.06)	1.06(1.05,1.06)	1.04(1.03,1.04)	1.03(1.03,1.04)
**Categorical variable**				
Q1	1.00	1.00	1.00	1.00
Q2	2.32(1.92,2.81)	2.29(1.88,2.77)	1.79(1.46,2.81)	1.76(1.43,2.16)
Q3	3.76(3.13,4.51)	3.72(3.10,4.46)	2.20(1.81,2.67)	2.06(1.69,2.52)
Q4	7.95(6.66,9.47)	7.96(6.66,9.51)	3.36(2.75,4.10)	2.91(2.37,3.58)
p for trend	<0.001	<0.001	<0.001	<0.001

*OR* odds ratio, *CI* confidential interval

Model 1 = adjusted for (age+gender+weight+race)

Model 2 = Model 2 + all Disease severity scoring system

Model 3 = Model 2+ (RRT+ Mechanical ventilition+hypertension+CKD+CLD+HF+neuropathy+malignancy+COPD+AKI)

### Kaplan-Meier survival curve analysis

**Fig. 2:**
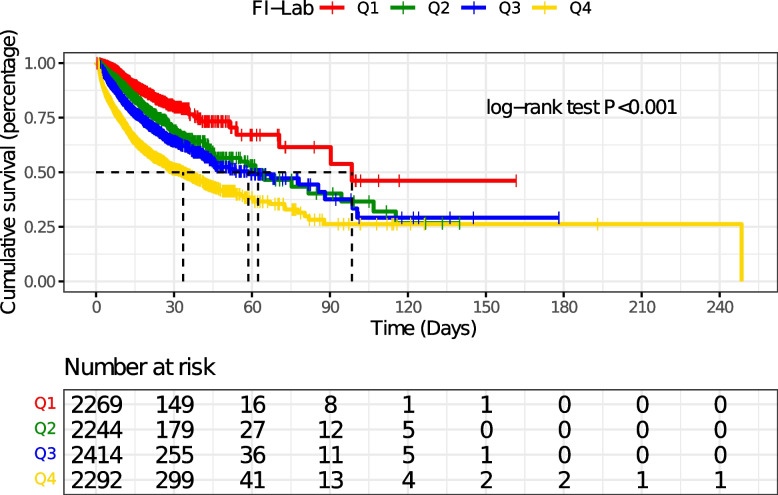
Kaplan-Meier survival curve of cumulative survival rate during hospitalization for groups

The Kaplan-Meier survival curves are shown in the Fig. 2, and after grouping by quartiles, the cumulative survival time during hospitalisation was significantly lower as FI-Lab increased (log-rank test, *P*<0.001).

### Subgroup analyses

**Fig. 3:**
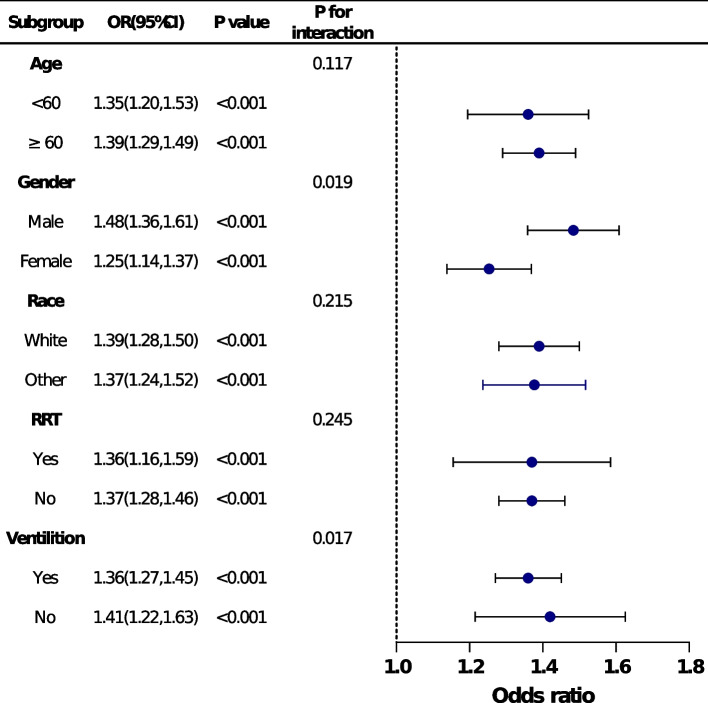
Subgroup analysis of the association between FI-Lab and in-hospital mortality. Each stratification adjusted for all the factors of model 3 in the Multivariable logistic regression, except for the stratification factor itself

In order to verify the robustness and consistency of our findings, we performed subgroup analyses to assess the association between FI-Lab and in-hospital mortality (Fig. 3). Overall, the positive association between FI-Lab and all-cause mortality during hospitalisation was generally consistent across subgroups, with higher FI-Lab associated with higher mortality. Statistically significant interactions were observed in the sex (*P*=0.019) and mechanical ventilation (*P*=0.017) subgroups. In ICU patients with septic shock, males and patients not receiving mechanical ventilation tended to have a higher risk of in-hospital death due to elevated FI-Lab than females and patients receiving mechanical ventilation.

### Non-Linear relationship between FI-Lab and in-hospital mortality

**Fig. 4:**
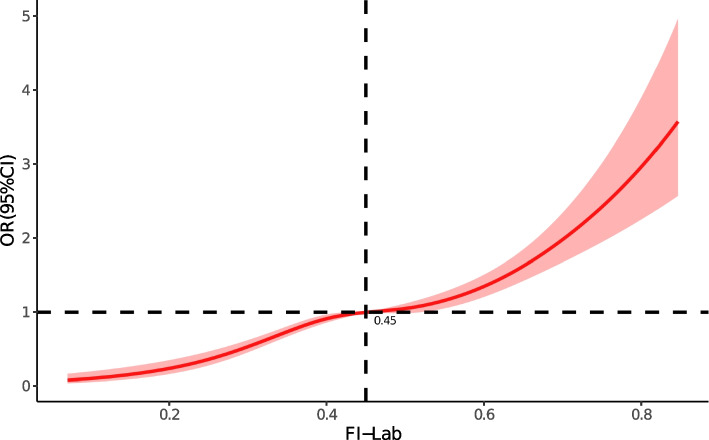
Spline curves showing the association of FI-Lab as a continuous variable with in-hospital mortality. Spline curves were adjusted for all the factors of model 3 in the Multivariable logistic regression

RCS showed that there was a non-linear relationship between FI-Lab at ICU admission and the risk of mortality during hospitalization in patients with septic shock (χ^2^= 15.25, *P *<0.001). When FI-Lab was 0.45, its OR was 1. Overall, with the increase of FI-Lab, the risk of mortality during hospitalization in patients with sepsis shock increased accordingly, as shown in Fig. 4.

## Discussion

To the best of our knowledge, this study is the first to explore the relationship between FI-Lab and in-hospital mortality in ICU patients with septic shock. In the present study, the FI-Lab index was higher in the non-surviving group of patients with sepsis compared with the surviving group, and Mortality during hospitalisation increased as FI-Lab rose. Subgroup analyses and Kaplan-Meier survival curves showed similar results.The RCS demonstrated a non-linear relationship between FI-Lab and the risk of in-hospital death, with an overall corresponding increase in the risk of death as the FI-Lab index increased. Therefore, when the FI-Lab index is significantly elevated in patients with septic shock in the ICU, it can be indicative of the patient’s prognosis during hospitalisation, and therefore more attention should be paid to this issue.

Frailty may provide additional patient prognostic information beyond age and standard risk factors, and one meta-study found that frailty was associated with in-hospital mortality in patients admitted to ICU (RR 1.71, 95%CI 1.43-2.05) [19]. Sepsis is often the final straw that crowds out vulnerable individuals. Exposure to inflammatory mediators and immune dysregulation due to infection are important pathophysiological reasons for the development of frailty [20, 21]. A recent multicenter prospective observational study demonstrated that in patients with sepsis when the Clinical Frailty Scale was used to define frailty, pre-existing frailty was associated with an increased rate of in-hospital mortality (adjusted OR 2.00, 95%CI 1.39-2.89) compared with patients without frailty [22]. In this study, the application of FI-lab to evaluate the prognosis of patients with septic shock in the ICU yielded consistent findings. We used propensity scores as balanced scores to adjust for confounding variables [23]. In our analyses, we found that FI-Lab was higher in patients who died in hospital from septic shock, and that the risk of in-hospital death increased as FI-Lab rose, even after adjusting for propensity scores and potential confounding variables. Frailty is not an irreversible condition and can be a potentially preventable and treatable disorder. Early detection and appropriate management of frailty, such as physical activity and nutritional supplementation, is therefore important for patients with comorbid sepsis and may help to improve the prognosis of patients [24–26].

The FI-lab scale is an objective measure of frailty with well-established testing techniques for the constituent parameters, developed and obtained from community-dwelling populations [11, 12]. Unlike Howlett et al., who used 33 items in the calculation of the FI-Lab, in the present study at least 21 items were used in the construction of the FI-Lab scale, and the minimum and maximum sizes of item books are unknown and require further exploration. Recent research has shown FI-Lab to be predictive of in-hospital mortality in critically ill ICU patients, and that its combination with other measures of frailty may improve the identification of critically ill patients at increased risk of in-hospital mortality [27]. In elderly patients with community-acquired pneumonia, YM et al. found FI-Lab to be a valid predictor of mortality at 30 days and has the potential to be efficacious as an adjunct to CRUB-65 (AUC 0.850, 95% CI: 0.809-0.892) and PSI (AUC 0.839, 95% CI: 0.794-0.885) [28]. In addition, FI-Lab can be added to disease severity scores to improve the ability to predict short-term and long-term mortality in patients with acute severe myocardial infarction (AMI) [16]. The complementary value of FI-Lab as a companion measure to other commonly used assessment measures is worthy of further exploration and research.

The strengths of our study are as follows. First, this study is the first to investigate the relationship between FI-Lab and in-hospital mortality in patients with septic shock in an intensive care unit. Second, we used data retrieved from the real-world MIMIC-IV database, have used PSM to reduce the effect of confounders, and the study findings are plausible. However, there are some shortcomings. To begin with, This was a retrospective study with data from electronic databases. About 18% patients with septic shock were excluded from the study due to a lack of necessary data, which could have biased the results. For example, the lack of information caused by patients’ varying severity of illness and the lack of widespread availability of blood tests can lead to sample selectivity bias. Furthermore, we only calculated the FI-Lab at the time of admission to the ICU, and the FI-Lab may change with the time or condition of the patient during hospitalization. The trend of FI-Lab can be obtained by the dynamic extraction calculation of the applet, and the relationship between this change and the prognosis of ICU septic patients is unclear and deserves to be further explored. Finally, we were not able to obtain information on long-term outcomes such as quality of life, disability, readmissions, and mortality in the long term.

## Conclusion

This current study demonstrates that elevated FI-Lab is strongly associated with poor prognosis during hospitalisation in ICU patients with septic shock. Frailty is not irreversible, for example, increased exercise and nutrition at the right time, and we need to focus on septic shock patients with higher FI-Lab on admission to the ICU.

### Supplementary Information

Supplementary Material 1. Reference range of items used for construction of FI-Lab.

## Data Availability

The datasets used and analysed during the current study available from the corresponding author on reasonable request. The details of the data screening codes for our analyses, which were provided by the authors of the MIMIC III and IV database, can be found at GitHub (https://github.com/MIT-LCP/mimic-code).
