# Supplementary material for: The association of a frailty index derived from laboratory tests and vital signs with clinical outcomes in critical care patients with septic shock: a retrospective study based on the MIMIC-IV database
Source: BMC Infect Dis. 2024 Jun 10;24:573. doi: 10.1186/s12879-024-09430-w (PMC11163768; doi:10.1186/s12879-024-09430-w)
Supplement: Supplementary file 1 — Supplementary Material 1. Reference range of items used for construction of FI-Lab. [file 12879_2024_9430_MOESM1_ESM.pdf]

Table 5 Reference range of items used for construction of FI-Lab Items

Items	Reference range
Vital signs	
Systolic blood pressure (mm Hg)	90–140
Diastolic blood pressure (mm Hg)	60–90
Heart rate (bpm)	60–99
Blood samples	
White cell count (×103/μL)	4–11
Platelet count (×109/L)	150–440
Hemoglobin (g/dL)	Female: 12–16<br>Male: 14–18
Total bilirubin (mg/dL)	0–1.5
Alanine transaminase (Units/L)	0–40
Albumin (g/dL)	3.5–5
Alkaline phosphatase (Units/L)	35–105
Lactate dehydrogenase (Units/L)	94–250
Urea nitrogen (mg/dL)	6–20
Creatinine (mg/dL)	Female: 0.4–1.1<br>Male: 3.5–5.2
Glucose (mg/dL)	70–110
Potassium (mmol/L)	3.5–5.4
Sodium (mmol/L)	133–145
Calcium (mg/dL)	8.4–10.3
Phosphorus (mg/dL)	2.7–4.5
Prothrombin time (s)	9.4–12.5
International normalized ratio	0.9–1.1
APTT (s)	25–35
Fibrinogen (mg/dL)	150–400
Troponin T (ng/mL)	0–0.01
Arterial blood gas samples	
PH	7.35–7.45
PO2 (mm Hg)	85–105
PCO2 (mm Hg)	35–45
Lactate (mmol/L)	0.5–2
Urine sample	
Leucocytes	Negative
Erythrocytes	Negative
Protein	Negative
Glucose	Negative
Ketones	Negative
Bilirubin	Negative

APTT, activated partial thromboplastin time; PH, potential of hydrogen; PO2, partial pressure of oxygen; PCO2, partial pressure of carbon dioxide
